# Legislation: California Enacts Safe Cosmetics Act

**DOI:** 10.1289/ehp.114-a402

**Published:** 2006-07

**Authors:** Cynthia Washam

Californians frustrated with what they consider the FDA’s loose control over cosmetic safety have taken matters into their own hands with the country’s first state cosmetics regulatory act, which takes effect in January 2007. The California Safe Cosmetics Act of 2005 will require manufacturers to report the use of potentially hazardous ingredients to the state Department of Health Services (DHS), which in turn will alert consumers. The DHS has the authority to investigate whether the product could be toxic under normal use and to require that manufacturers submit health effects data. Manufacturers that continue marketing products deemed unsafe in California could face legal action.

“The legislation’s sponsors believe that the basis of the law is the public’s right to know,” says Kevin Reilly, DHS deputy director of prevention services. The new law uses the list of toxicants drawn up under California’s Proposition 65, which mandates that the governor publish a list, updated at least yearly, of chemicals that are known to the state of California to cause cancer, birth defects, or other reproductive harm.

Although the new act applies only in California, its effects are likely to reverberate nationwide. Consumer advocates predict that manufacturers seeking to avoid negative publicity will remove, rather than report, suspect ingredients. Those formulas would then be marketed coast to coast.

Impetus for the law stems from consumers’ concerns over long-term exposure to certain cosmetic ingredients. Cosmetic use has not been linked to chronic illnesses, but some products do contain carcinogens (such as formaldehyde, used in nail treatments), teratogens (such as lead acetate, used in two hair dyes), and other reproductive toxicants (such as di-*n*-butyl phthalate, used in nail treatments and dandruff shampoos).

Studies in recent years have shown that humans absorb and inhale sometimes surprisingly high levels of toiletry ingredients. In the November 2005 issue of *EHP*, a team led by Susan M. Duty of the Harvard School of Public Health demonstrated that urine concentrations of phthalate metabolites increased by 33% with each personal care product—hair gel or spray, lotion, deodorant, cologne, aftershave—that subjects used.

Historically, cosmetics safety has been in the hands of manufacturers; the FDA requires no premarket testing. Each year, an expert panel convened by the industry-funded Cosmetic Ingredient Review (CIR) identifies priority ingredients for which it conducts literature reviews and analyses to determine safety. The panel—made up of independent academic researchers and representatives from industry, consumer interests, and the FDA—has declared 9 of the 1,286 ingredients reviewed since 1976 unsafe for normal cosmetic use. But manufacturers are not obligated to eliminate any ingredients—at least one ingredient identified as unsafe by CIR, hydroxyanisole, is still used.

Safety advocates see evidence of any harm in any use as reason enough for a ban. “Ingredients suspected of causing cancer shouldn’t be used in cosmetics,” says spokesman Kevin Donegan of the Breast Cancer Fund, a San Francisco–based nonprofit that promoted the California bill.

F. Alan Andersen, director and scientific coordinator of CIR, counters that the dose creates the danger. “We don’t subscribe to the notion that if there’s ever an adverse effect, [a chemical] must not be in a product people use,” he says. “It doesn’t make sense to us to apply the precautionary principle. Instead, we use a risk assessment approach, and the wide margins of safety that we have found for chemicals such as phthalates using this approach assure us that actual use of cosmetics is safe.”

The law drew fierce opposition from individual companies and the Cosmetic, Toiletry, and Fragrance Association (CTFA) as it worked its way through the California legislature. “CTFA supports strong federal regulation by the FDA,” says Kathleen Dezio, executive vice president of public affairs and communications for the association. “For this reason, CTFA has generally opposed state-specific legislation that would undermine this national approach and lead to an unworkable state-by-state patchwork of rules . . . or unjustified, extreme requirements that are well beyond those placed on any other category of food, beverages, drugs, or consumer products.” She adds that CTFA has met with the DHS and “pledged our cooperation in accomplishing the requirements” of the law.

Some manufacturers have already ceded to public pleas for safer products. In the past two years, almost 350 of them signed a pledge promoted by the Campaign for Safe Cosmetics, a coalition of health and environmental groups, to use no chemicals linked to cancer or birth defects. Industry leaders L’Oréal and Revlon broke new ground last year when they promised that products they sold in the United States would meet more stringent European Union standards. In 2004 Europe enacted a ban on suspected carcinogens, mutagens, and reproductive toxicants in personal care products.

“We’re definitely seeing a shift in the attitude of manufacturers,” Donegan says. “They’re starting to see the benefits of removing anything that could cause cancer.”

## Figures and Tables

**Figure:**
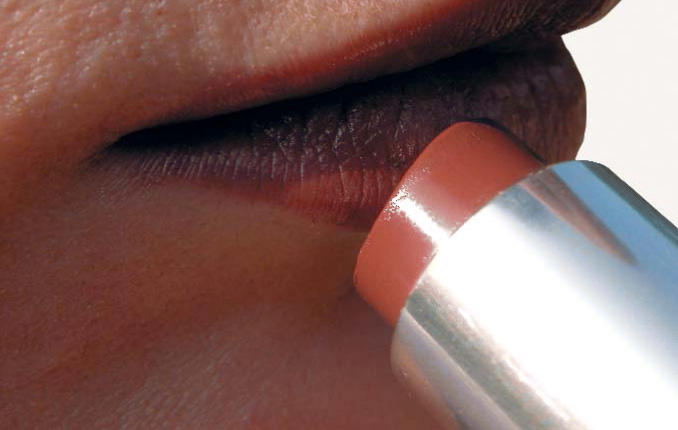
A safer smooch California recently enacted legislation that will require manufacturers to report potentially hazardous ingredients used in cosmetic and personal care products.

